# Modeling the growth dynamics of multiple *Escherichia coli* strains in the pig intestine following intramuscular ampicillin treatment

**DOI:** 10.1186/s12866-016-0823-3

**Published:** 2016-09-06

**Authors:** Amais Ahmad, Camilla Zachariasen, Lasse Engbo Christiansen, Kaare Græsbøll, Nils Toft, Louise Matthews, Søren Saxmose Nielsen, John Elmerdahl Olsen

**Affiliations:** 1 Department of Large Animal Sciences, Faculty of Health and Medical Sciences, University of Copenhagen, Frederiksberg C, Denmark; 2 Department of Veterinary Disease Biology, Faculty of Health and Medical Sciences, University of Copenhagen, Frederiksberg C, Denmark; 3 Department of Applied Mathematics and Computer Science, Technical University of Denmark, Lyngby, Denmark; 4 National Veterinary Institute, Technical University of Denmark, Frederiksberg C, Denmark; 5 Boyd Orr Centre for Population and Ecosystem Health, College of Medical, Veterinary and Life Sciences, University of Glasgow, Glasgow, UK

**Keywords:** Antimicrobial resistance, Ampicillin, Pharmacodynamic, Dosing strategies, Pig

## Abstract

**Background:**

This study evaluated how dosing regimen for intramuscularly-administered ampicillin, composition of *Escherichia coli* strains with regard to ampicillin susceptibility, and excretion of bacteria from the intestine affected the level of resistance among *Escherichia coli* strains in the intestine of nursery pigs. It also examined the dynamics of the composition of bacterial strains during and after the treatment. The growth responses of strains to ampicillin concentrations were determined using in vitro growth curves. Using these results as input data, growth predictions were generated using a mathematical model to simulate the competitive growth of *E. coli* strains in a pig intestine under specified plasma concentration profiles of ampicillin.

**Results:**

In vitro growth results demonstrated that the resistant strains did not carry a fitness cost for their resistance, and that the most susceptible strains were more affected by increasing concentrations of antibiotics that the rest of the strains. The modeling revealed that short treatment duration resulted in lower levels of resistance and that dosing frequency did not substantially influence the growth of resistant strains. Resistance levels were found to be sensitive to the number of competing strains, and this effect was enhanced by longer duration of treatment. High excretion of bacteria from the intestine favored resistant strains over sensitive strains, but at the same time it resulted in a faster return to pre-treatment levels after the treatment ended. When the duration of high excretion was set to be limited to the treatment time (i.e. the treatment was assumed to result in a cure of diarrhea) resistant strains required longer time to reach the previous level.

**Conclusion:**

No fitness cost was found to be associated with ampicillin resistance in *E. coli.* Besides dosing factors, epidemiological factors (such as number of competing strains and bacterial excretion) influenced resistance development and need to be considered further in relation to optimal treatment strategies. The modeling approach used in the study is generic, and could be used for prediction of the effect of treatment with other drugs and other administration routes for effect on resistance development in the intestine of pigs.

**Electronic supplementary material:**

The online version of this article (doi:10.1186/s12866-016-0823-3) contains supplementary material, which is available to authorized users.

## Background

Antimicrobial resistance threatens the efficacy of available antibacterial drugs. High resistance levels are directly linked with excessive and routine antimicrobial use, and antimicrobial exposure in food production animals contributes significantly to increased antimicrobial resistance [1]. Limiting the use of antimicrobials can reduce the prevalence of resistance. However, treatment of production animals cannot be avoided for animal welfare and economic purposes. It is therefore important to find ways to reduce resistance, while maintaining the capacity to treat diseased animals.

During treatment of infection, antimicrobial pressure affects non-target commensal bacteria. This disturbance may create large pools of resistant bacteria that could lead to a general increase and spread of antimicrobial resistance [2]. The major organ of concern is the intestine, since it contains the highest number of bacteria at risk, and confers a means of direct transfer of resistant bacteria via feces.

A possible way to reduce antimicrobial use is to treat animals individually by injection treatment, rather than using flock treatment, and this is commonly used for especially respiratory and systemic diseases in the pig industry. In doing so, the intestinal flora will be newer-the-less be subjected to selection by the antibiotic, and it is thus important to determine how best to reduce this selection pressure. This study specifically examines how individual treatment of pigs by injection with ampicillin affects the levels of resistance in commensal *Escherichia coli* in the intestines of pigs, using a modeling approach.

*E. coli* are commensal bacteria commonly used as indicators of antimicrobial resistance in animals, humans and food products [3–5]. They readily exchange antibiotic-resistant genes, not only between strains of the species, but also with other bacteria including those pathogenic to humans [6], and their resistance levels in livestock herds generally reflects the overall antimicrobial pressure in these herds [7]. In Denmark, an average of 29 % of *E. coli* isolates collected from healthy pigs and 33 % of pork isolates from slaughterhouse samples were found to be resistant to ampicillin in 2012 [8].

In simple terms, antimicrobial pressure favors a resistant population because the susceptible population is inhibited or killed by antimicrobial activity. However, the relationship between antibiotic concentrations, the natural variation in sensitivity in the bacterial population, and the outcome in terms of strain selection is very complex, which makes it difficult to study this problem using a traditional experimental approach.

Mathematical modeling techniques are frequently used to investigate antibiotic resistance [9]. Such models are based on in vitro and in vivo studies, and allow rapid analysis of dosing strategies to help overcome the problem of emergence of antimicrobial resistance [10]. Most modeling studies for optimizing treatment strategies have been based on point estimates (e.g. minimum inhibitory concentration (MIC), maximum drug concentration (*C*_*max*_), and area under the concentration time curve (AUC)) for a few strains of bacteria [11–13]. In contrast, newer models include the full time-course of pharmacokinetic (PK) and pharmacodynamic (PD) aspects, providing greater insight and a more accurate description of antimicrobial dosing strategies [14–19]. However, even these studies have been based on very few (sometimes only one) clinical bacterial strains, providing a poor reflection of the within-host bacterial dynamics. Pigs are co-colonized with a number of commensal *E. coli* strains, both sensitive and resistant ones, and there is a need to develop models that encompass the growth response of a more realistic strain collection, to obtain modeling results that better reflect real pig production systems.

Mathematical models used so far have not accounted for differences in the number of competing strains, or in the composition of strains with different susceptibilities, on the effect of a given treatment strategy [20–22]. In contrast, in the present study, we have specifically modeled the effect of strain composition and number on the selection of resistant bacteria after ampicillin intramuscular (IM) treatment. Our primary objectives were to: 1) characterize the growth response of 50 *E. coli* strains when treated with ampicillin over a wide concentration range based on a PD model; 2) predict the competitive growth of randomly selected *E. coli* strains in a pig under drug exposure based on in vivo PK data from ampicillin IM treatment; 3) assess the optimum dose level, dosing frequency and treatment duration to suppress or delay the growth of resistant strains; and 4) analyze the importance of bacterial excretion (diarrhea), and number of resistant bacteria prior to treatment, on the buildup of resistance.

## Results

The aim of this study was to determine how different treatment strategies and characters of the intestinal flora of pigs affected the growth of ampicillin-resistant and sensitive strains following IM treatment with ampicillin. To achieve this aim, the study comprised three steps: 1) estimation of pharmacodynamic parameters of a representative collection of porcine *E. coli* strains based on in vitro growth experiments, 2) estimation of concentration-time profiles of ampicillin following IM treatment, based on literature data, and 3) predictions of the competitive growth of multiple *E. coli* strains with varying duration and frequency of IM ampicillin treatment and with varying excretion rates of bacteria from the intestine, corresponding to the effect of diarrhea.

### In vitro growth response of *E. coli* strains to ampicillin

**Fig. 1:**
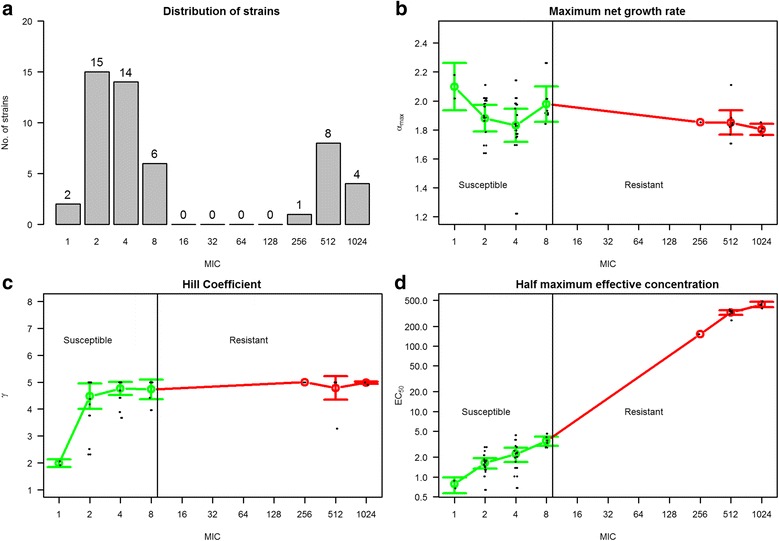
**a** Distribution of strains according to MIC values. **b**, **c**, **d** The three PD parameters for the 50 *E. coli* strains versus their MIC values shown as means with 95 % confidence intervals. The green line represents susceptible strains and the red line represents resistant strains

Fifty *E. coli* strains were randomly selected from the National Antimicrobial Resistance monitoring program to represent the population of commensal *E. coli* from pigs in Denmark [23]*.* These strains were used as a foundation to study the effect of ampicillin on the growth dynamics of sensitive and resistant strains. Among the randomly chosen strains, 13 (26 %) were found to be resistant to ampicillin with an MIC ≥ 32 μg/ml (Fig. 1a). The strains were exposed to ampicillin in triplicate at a range of constant concentrations from 0.06 to 256 μg/ml (see Methods). Single net bacterial growth rate at corresponding concentration was estimated from the growth curves, using an exponential model fit to triplicates as repeated measures. These estimated rates were assessed using fit curve and confidence intervals (data not shown). A relationship between ampicillin concentrations and net bacterial growth rates was obtained for each strain, and an *E*_*max*_ model was fitted to this relationship based on three PD parameters.

No relation was found between the MIC of the strain and net growth rates in the absence of a drug (maximum growth rate, *α*_*max*_), and the mean growth rate of susceptible and resistant strains did not differ significantly, indicating that the resistant strains did not carry a fitness cost for their resistance (Fig. 1b). The hill coefficients (*γ*), i.e. the steepness of the response curve for each strain with growing concentration of antibiotic, were also found not to differ significantly between the two groups of susceptible and resistant strains, however, the most susceptible strains with MIC <2 ug/ml apparently were more affected by increasing concentrations of antibiotics that the rest of the strains (Fig. 1c). A strong linear relationship was found between MIC and *EC*_*50*_ on a log-log scale, showing a relation between the point estimate and the dynamic activity of the drug (Fig. 1d). These parameters capture the dynamic relationship between antimicrobial concentration and net growth rate and should be considered along with MIC when evaluating treatment strategies. These parameters were used in the mathematical model to predict in vivo growth.

### Pharmacodynamics of ampicillin in pigs following IM ampicillin treatment

The plasma concentration profile in pigs after an IM ampicillin treatment was obtained from literature and was fitted to a two-compartment model [24]. Different concentration profiles were simulated using estimated transfer rates based on the combinations of dosing factors. In all of the simulated profiles, concentrations remained below the cut-off value between resistant and susceptible strains (16 μg/ml) in the intestine, due to fast elimination from the body, indicating a poor likelihood that IM ampicillin treatment can be used to eliminate the more resistant part of the commensal *E. coli* flora, even if concentrations were increased above the recommended dose (data not shown).

### Modeling the effect of IM treatment with ampicillin on dynamics of commensal *E. coli* in the intestine of pigs

To model the effect of treatment on the growth of sensitive and resistant strains, 12 strains (four of which were resistant to ampicillin) and their characteristics were randomly selected among the 50 strains as inhabitant of the intestine in pigs treated IM with ampicillin. Growth dynamics of strains before, during and after treatment was measured in four different ways (Additional file 1: Figure S1: Growth of individual strains (Additional file 1: Figure S1, top-left), the sum of susceptible and resistant counts (Additional file 1: Figure S1, top-right), a 95 % simulation envelope around the mean value from 100 repeats, (Additional file 1: Figure S1, bottom-left), and in fractions of mean values of resistant and susceptible bacteria (Additional file 1: Figure S1, bottom-right).

**Fig. 2:**
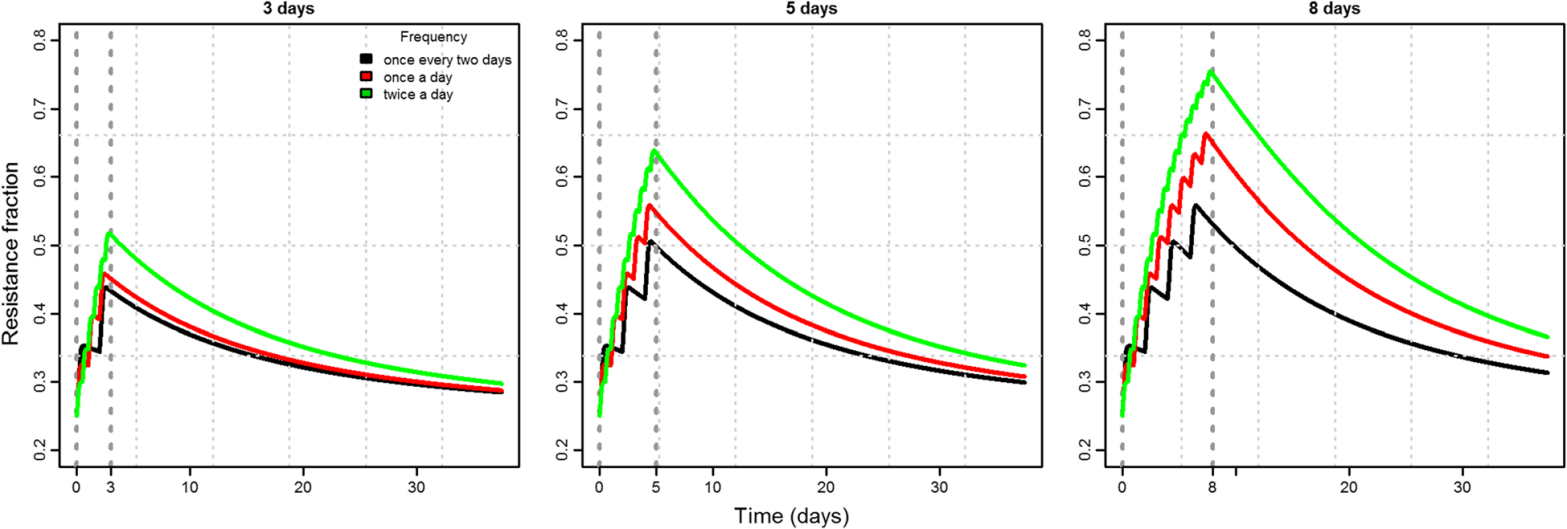
Total resistance fraction over time for a composition of 12 strains at varying treatment frequencies (colours) and durations (sub-plots). The treatment window is shown by the vertical dotted lines

**Fig. 3:**
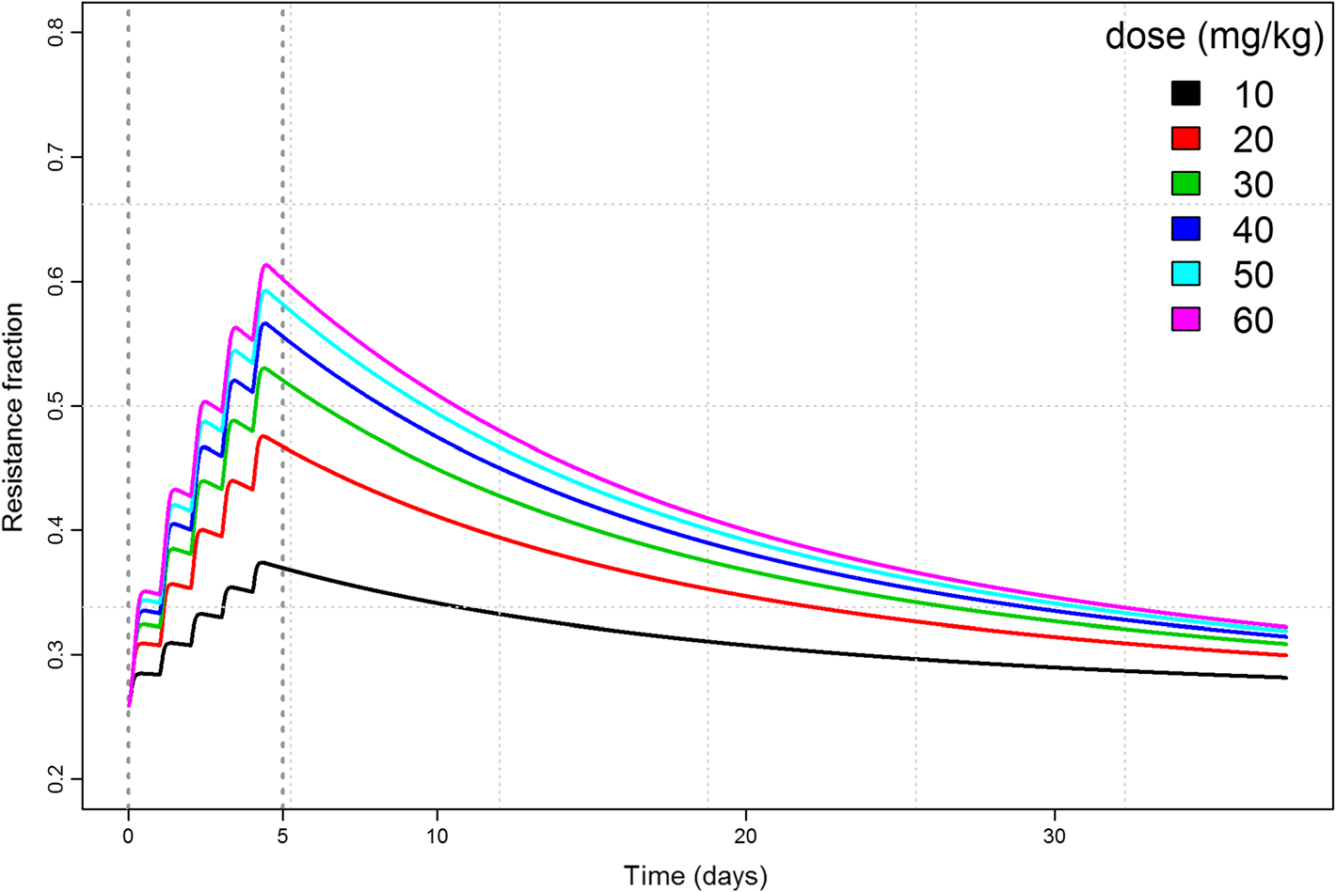
Total resistance fraction over time for competitive growth of 12 strains at different daily doses (colours) of ampicillin IM treatment. A 5-day long treatment duration is shown as vertical dotted lines with one treatment per day

Among the treatment-combinations tested, the modeling revealed that short treatment duration resulted in the lowest fraction of resistant strains after treatment and, consequently, a faster return to equilibrium (Fig. 2). The effect of dosing frequency (with fixed amount of antibiotic) on the fraction of resistance was found to be low, however, the differences increased with longer treatment durations (Fig. 2). A summary of the predicted resistance fractions at three time points for the tested treatment strategies can be seen from the (Additional file 1: Figure S2). The effect of increased daily dose was also modelled, resulting in higher resistance levels with higher daily doses (Fig. 3).

**Fig. 4:**
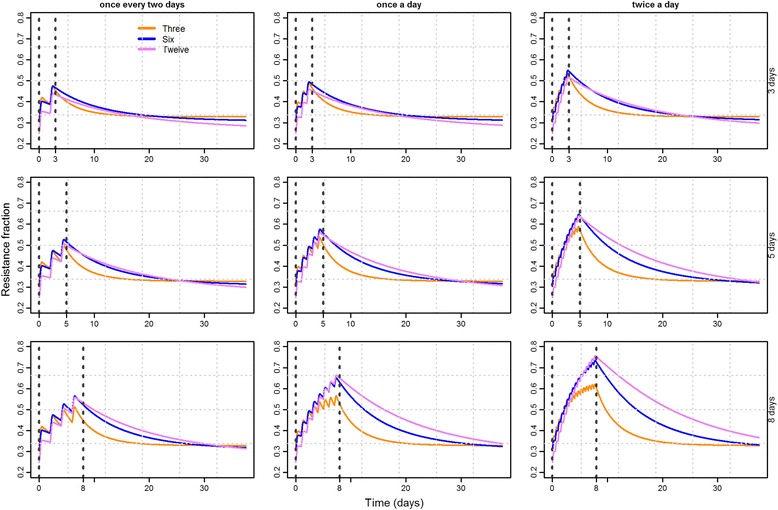
Fraction of total resistant counts over time for nine combinations of dosing factors where different colors represent the number of competing strains K. Vertical lines depict the treatment windows. Each row of the panel represents different treatment durations (3, 5, 8 days) and each column of the panel represents changing dosing frequencies (once every 2 days, once a day, twice a day)

Besides dosing factors, other parameters may have an effect on the growth of resistant bacteria and could play an important role when deciding upon optimal treatment strategies, and uniquely our modeling incorporated such factors in the modeling scenarios. The number of different commensal *E. coli* harbored by each pig may vary considerably [25], and to investigate the importance of this for resistance development, we modeled growth dynamics in the pig intestine with different numbers of competing strains. The number of strains was found only to influence on resistance levels following long treatment duration, while it was not important for shorter treatment times (Fig. 4). In addition, for longer treatment durations, the simulations revealed a tendency that pigs with fewer strains more quickly returned to the pre-treatment equilibrium in the intestine (Fig. 4).

**Fig. 5:**
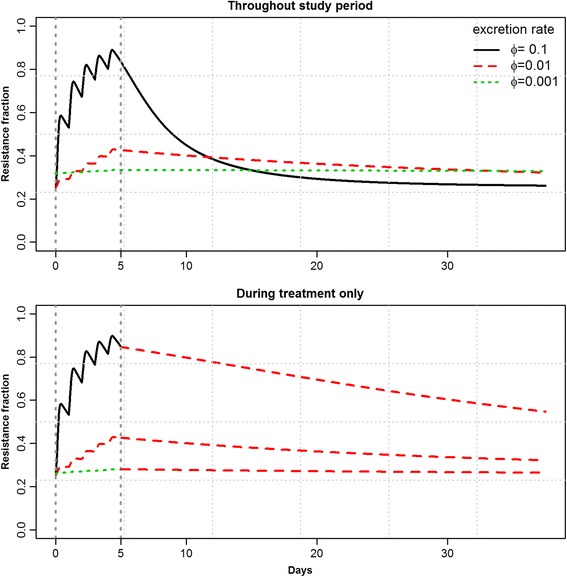
Mean resistant fraction of bacterial counts for 100 model repeats with varying outflow rate. Outflow rates were first kept constant throughout the simulated time (*top*), and secondly set to 0.01 outside treatment time (*bottom*)

Pigs with diarrhea can be assumed to excrete bacteria, including *E. coli* in larger quantities than pigs without diarrhea. To investigate the importance of this phenomenon, the model incorporated the excretion of a proportion of the bacterial population. When this was included, higher resistance levels were found for higher outflow rates during treatment (Fig. 5), yet resistance dropped faster post-treatment (Fig. 5, *top*). However, if the increased outflow rate was limited to the treatment period (corresponding to a situation where the treatment results in a cure), the opposite was observed: resistance levels dropped more slowly after the treatment end (Fig. 5, *bottom*).

## Discussion

The current study modeled the effect of IM ampicillin-treatment strategies in pigs on the balance between sensitive and resistant *E. coli* strains in the intestine of the pig, in order to be able to recommend improved treatment strategies that can be further elaborated. To compare treatment strategies by field-testing is very cumbersome, and our approach therefor constitutes a shortcut to better treatment strategies. It must be stressed that further investigations with the treatment strategies that revealed the least resistance development should be carried out.

Our model simulated the competition between multiple bacterial strains (including both susceptible and resistant ones) representative of the Danish natural population of pig *E. coli*. In contrast to previous studies [17, 19–22], we used dynamic plasma concentration profiles of a drug, as opposed to the use of static PK parameters, we based our modeling on growth parameters on a representative collection of naturally occurring porcine, commensal *E. coli* strains, we modeled resistance development taking into account that different pigs may harbor different numbers of commensal *E. coli,* and we included the fact that some pigs may shed bacteria in higher numbers due to diarrhea in our modeling. Since the approach taken in genuine, it sets a model for future modeling studies with other antibiotics and other livestock species.

Growth curves were obtained using the BioScreen format, which allowed us to expose many strains to a range of static concentrations of ampicillin to create input data for our modeling, rather than the small number possible in manual growth experiments. We did not observe a fitness cost of carrying ampicillin resistance in the current study. Although some of the resistant strains were found to have a reduced growth rate compared to susceptible strains, there was no overall difference in growth between the susceptible and resistant groups, which suggests that the fitness cost associated with the resistance mechanisms in our strains was small. We have not characterized the mechanism responsible for ampicillin resistance in our study, and further studies are needed to elucidate whether the observation of low fitness cost is associated with one or more resistance mechanisms. Commensal *E. coli* strains from pigs in Denmark are commonly resistant to tetracycline, and we have previously shown a lack of fitness cost associated with resistance to this drug [23, 25]. In contrast to the growth rates, a linear relation between *EC*_*50*_ and MICs on the log-log scale was observed. This indicates that the difference between strains in their ability to degrade ampicillin is not dependent on the ampicillin concentration—an observation that deserves further attention.

Due to the high number of strains characterized, the parameters used as input to our model study are more epidemiologically realistic than those from previous studies, which based growth modeling on a limited number of strains, mostly of clinical origin [26–29]. Coexistence of multiple strains in our model was achieved by using a double growth restriction term as also used in our recently published studies [24, 30]. The double growth restriction term enables a dynamically balanced system where no strain conquers the system. Previous studies used single growth restriction terms. This can lead to a situation where one strain with a high growth rate outgrows all other strains, and it is not possible for equilibrium to be established. A biological interpretation of the double growth restriction is that the total population cannot exceed the carrying capacity due to the limited availability of nutrients and space, and the full resource cannot be used by a single strain, since it will alter the growth conditions when it removes the nutrients on which its superior growth abilities are based. Conjugation or other modes of transfer of resistance were not included in the model, as the impact of such events was assumed to be generally unimportant given the previously established resistant population in modern pig production [31].

The modeling found that short treatment duration resulted in less selection of resistant bacteria than longer durations. This finding is in agreement with a previous modeling study [22]. The most logical explanation is that this is the case because this strategy gives resistant strains less time to outcompete susceptible strains under sub-MIC concentrations. Only a very small variation in resistance was observed for different dosing frequencies (the same amount of drug dispersed over different lengths of time). We also observed that the number of competing strains only had a very small effect on the final proportion of resistant strains, when using short treatment duration. In contrast, for longer treatment durations, higher resistance levels were observed with increased number of resistant strains. Together, these results indicate that provided the treatment is clinically effective, it will always be an advantage to keep the duration of treatment as short as possible. Based on these results, it would be valuable to test different strategies for ampicillin with short-duration treatment in field-testing in pigs, in order to support recommendations for new treatment protocols.

No in vivo studies on the importance of increased bacterial excretion, for example as a consequence of diarrhea, on resistance development has been carried out. One modeling based study has indicated that the spread of resistant bacteria between pigs plays an important role in the balance between resistant and sensitive strains following treatment, favoring resistant strains [30]. In the current study, the outflow rate was also found to influence the proportion of resistant strains. When diarrhea was assumed to disappear at the end of treatment, the higher outflow rate during treatment resulted in higher numbers of resistant bacteria, while continuous diarrhea was associated with a rapid return of resistant bacteria after treatment. Thus, bacterial excretion appears to drive the speed at which the system equilibrates. Whether this occurs in reality remains to be investigated, but among other factors, it will depend on whether the different strains have the same ability to adhere to the intestine. As mentioned, a previous modeling study suggested that increased outflow will tend to favor resistant strains for groups of more than one pig following oral treatment with tetracycline [30], and this factor has to be considered together with our observations to have the full picture.

## Conclusions

Our model predictions showed that short treatment duration with ampicillin (IM) will result in fewer resistant *E. coli* in the intestine of the treated animal and a faster return to equilibrium. The effect of dosing frequency was higher for longer treatment durations. Besides dosing factors, the epidemiological factors (such as number of competing strains and bacterial excretion) need to be considered further before designing optimal treatment strategies. The modeling approach used in the study is generic, and could be used for evaluation of the effect of treatment with other drugs and other administration routes for predicting resistance levels in pigs.

## Methods

### Pharmacodynamics

As part of the DANMAP 2010 report, 160 *E. coli* strains were collected from fecal samples of healthy pigs at slaughter [32]. From these, a total of 50 *E. coli* strains were randomly selected for in vitro experiments. The 50 *E. coli* isolates had previously been subcultured and confirmed as *E. coli* by biochemical tests (lactose and indole test) and MALDI-TOF MS analysis [23]. The susceptibility towards ampicillin was tested by broth microdilution according to the CLSI standards [33]. Two-fold dilutions of ampicillin sodium salt (Sigma-Aldrich, Switzerland) from 0.125 to 1024 μg/ml were prepared in Cation-Adjusted Müllel-Hinton Broth (CAMHB) and distributed in microtitre plates. CAMHB without ampicillin were included to serve as positive growth controls. The plates were inoculated with dilutions of 0.5 McFarland turbidity standard adjusted bacterial saline suspensions, prepared from overnight cultures on blood agar. Final bacterial concentration in each well was approximately 5 × 10^5^ CFU/ml. The *E. coli* reference strain *E. coli* ATCC25922 was used as quality control. Strains with MIC ≤ 8 μg/mL were considered to be ampicillin susceptible, and strains with MIC ≥ 32 μg/mL were considered ampicillin resistant [34].

Growth curves for the effect of ampicillin on the 50 *E. coli* strains were performed using the automated microbiology growth curve analysis system BioScreen C™ (Oy Growth Curves Ab Ltd, Finland). All isolates were grown without antibiotics, as well as with two-fold dilutions of ampicillin sodium salt; ampicillin susceptible isolates were grown in two-fold dilutions from 0.06 to 16 μg/mL ampicillin, and resistant isolates in two-fold dilutions from 1 to 256 μg/mL ampicillin. Ampicillin suspensions and bacterial inoculum were prepared as described above. The final volume in each well was 200 μl, with a final inoculum concentration of approximately 5 × 10^4^ CFU/ml. Blank samples of all ampicillin dilutions as negative growth controls, as well as the *E. coli* ATCC25922 strain for plate-to-plate comparisons, were included on all plates. The plates were placed in the BioScreen and incubated at 37 °C with continuous shaking, and optical density (OD) at a wavelength of 600 nm was measured every 5 min for 18 h. All BioScreen growth experiments were performed in biological triplicates. For the strains with an MIC greater than the treated ampicillin concentrations, no growth was considered at a concentration equal to or above the MIC.

Raw data from the BioScreen were extracted in Microsoft Excel. OD values of the blank samples were subtracted from the measured OD values of the samples at their respective ampicillin concentration and time point. In this study, the maximum reliable OD value for which a linear relationship between OD and CFU was considered valid was set as 0.1. An exponential equation (eq. 1) was fitted to the growth curve below the OD value of 0.1 to estimate the net bacterial growth rates at corresponding ampicillin concentrations using a nonlinear least square algorithm nls() function of the R software (version 3.1.2 for windows) [35].

1 $$ {Y}_t=\mu {e}^{\alpha t}+\beta +{\varepsilon}_t $$

Where *Y*_*t*_ is the OD value, *μ* is the initial OD value at time zero, *⍺* is the growth rate, *β* is an offset variable for the adjustment of *μ,* and *ε*_*t*_ a normal error with mean zero and constant variance *σ*^2^; i.e., *ϵ*_*t*_ = N(0, *σ*^2^).

The relationship between ampicillin concentrations and the estimated net bacterial growth rates was established and analyzed using the three parameter *E*_*max*_ model (known as the Hill equation) as below:

2 $$ a(c)={a}_{\max }-\frac{a_{\max }{\left(\frac{c}{E{C}_{50}}\right)}^{\gamma }}{1+{\left(\frac{c}{E{C}_{50}}\right)}^{\gamma }} $$

where *a*_max_ is the bacterial growth rate in the absence of the drug (maximum effect); *EC*_50_ is the concentration at which the drug effect is reduced to 50 %; and γ denotes the Hill coefficient, which is the measure of the steepness of the sigmoid relationship between concentration *c* and the growth rate at concentration *c*. The PD model parameters *a*_max_ and γ were compared for susceptible and resistant strains using the Wilcoxon rank-sum test in R and the linear relation between MIC and *EC*_50_ was analyzed using the lm() function in R.

### Pharmacokinetics

The mean plasma concentrations of ampicillin after IM treatment in pigs was taken from the literature [36]. A two-compartmental PK model was fitted to the values of the plasma concentration and time profile to estimate the transfer rates between two compartments. The two compartments included a central compartment and a peripheral compartment as previously described [24], but fitted with ampicillin. Based on the estimated rates, different concentration-time profiles for different combinations of treatment durations and dosing frequencies delivering the same daily dose of 40 mg/kg were simulated. These different concentration-profiles were incorporated into the model as described in the next section. Moreover, to investigate the effect of different daily doses, concentration-time profiles were incorporated into the model for a range of daily doses of (10, 20, 30, 40, 50, 60) mg/kg.

### The PK-PD Model

We modeled the changes in the bacterial counts of individual bacterial strains in a pig using growth parameters from the PD model, combined with the in vivo drug profile using an ordinary differential equation [24] given by:

3 $$ \frac{d{N}_i}{dt}={\alpha}_{\max, \mathrm{i}}\left(1-\frac{c^{\gamma_i}}{c^{\gamma_i}+E{C}_{50,i}^{\gamma_i}}\right)\left(\frac{N_{max}-{N}_i}{N_{max}}\right)\left(\frac{N_{max}-{\displaystyle \sum }{N}_i}{N_{max}}\right){N}_i-\varphi {N}_i $$

The left hand side of the equation shows the changes in the bacterial counts *N*_*i*_ of strain *i*. The first bracketed term on the right hand side of the equation gives the drug efficacy as a function of the three PD model parameters (*a*_max_, *EC*_50_, *γ*), and the in vivo drug concentration-time profile *c.* The remaining bracketed terms are density-dependent limitations to growth, which depend on the carrying capacity, *N*_*max*_, and the total bacterial counts summed over all competing strains in the pig, *ΣN*_*i*_. The final term is the excretion of strains from a pig with an outflow rate $\varphi$ [30].

Firstly, 12 *E. coli* strains were randomly drawn from the 50 strains, along with their PD parameters. The model was allowed to run without antimicrobial treatment with these 12 strains competing in the pig intestine in order to establish dynamic equilibrium (Additional file 1: Figure S3, without treatment). The dynamic equilibrium was defined as the stage at which multiple strains co-exist with very small changes over a long period of time, in the absence of the drug. During the treatment period, however, antimicrobial pressure temporarily disturbs the equilibrium (Additional file 1: Figure S3, with treatment). Strains would return to a state of equilibrium when treatment stopped, but could differ from the pre-treatment equilibrium. A dynamic equilibrium could only be established in our model when the outflow rate was less than the maximum growth rate of the individual strains (i.e. *φ < α*_*max*_).

Different treatments were introduced once the equilibrium was attained, with the first treatment day denoted as Day 0 (Additional file 1: Figure S3). The model was allowed to run for a period of 35 days to assess the effect of dosing factors on the post-treatment growth dynamics. To capture the variation in growth characteristics and susceptibility levels in the mix of strains, this was repeated 100 times with 12 *E. coli* strains*.* In each repeat, 12 different strains were randomly drawn from 50 *E. coli* strains. To assess the influence of the number of competing strains, model predictions were also made with the inclusion of only six and then three competing strains.

A homogenous distribution of the bacterial population was assumed within the intestine of a pig [37]. A plasma concentration profile was used to represent the bacteria-drug interaction after an IM injection, as plasma concentration is often used as a surrogate for the concentration at the site of interaction, which in this case is the intestine [38]. To capture the effect of excretion, the outflow rate was varied both during the treatment as well as throughout the simulation period.

The carrying capacity *N*_*max*_ was set to 10^10^ bacteria. An outflow rate, *φ*, of 0.01 per hour was taken from a published experimental study that estimated the hourly fractional inflow and outflow of *E. coli* “free-living” in the large intestine from an in vivo study in post-weaned dairy calves [20, 39]. The initial numbers of individual strains were randomly selected in the interval 10^6^ to 10^9^ with the total sum not exceeding the carrying capacity of 10^10^. A seed was set before the model run to allow runs with the same strains and initial values under different treatment protocols to be compared. As around 30 % of detected *E. coli* were ampicillin-resistant in the Danish surveillance scheme, competing strains were selected with a proportion of one third (i.e., 4, 2 or 1) resistant [8_ENREF_30]. The model was written in R (version 3.1.2 for windows) [35], and all data were analyzed and graphed using R.

## Additional file

Additional file 1: Figure S1. Bacterial counts over time for 12 individual competing strains in a pig intestine, represented by different colours (top-left). Sum of susceptible (black) and resistant (red) counts (top right). Mean with 95 % simulation envelope from 100 model repeats (bottom left). Mean fraction of resistant counts over time (bottom right). **Figure S2**. Mean resistance fraction with 95 % simulation error bars from 100 model repeats at three different time points: day 0, day max and day 35. Day max represents the day with the maximum resistance. Different colours represent dosing frequencies and the columns represent treatment durations. **Figure S3**. Bacterial counts over time for 12 competing strains (different colours) with different growth characteristics without treatment (left), and with treatment (right). Vertical dotted lines indicate treatment duration. (PDF 466 kb)

## Abbreviations

AUC: Area under curve
CAMHB: Cation-Adjusted Müllel-Hinton Broth
CFU: Colony forming units
*E. coli*: *Escherichia coli*
IM: Intramuscular
MH-2: Mueller-Hinton 2
MIC: Minimum inhibitory concentration
OD: Optical density
PD: Pharmacodynamic
PK: Pharmacokinetic
