# Supplementary material for: Modeling the growth dynamics of multiple Escherichia coli strains in the pig intestine following intramuscular ampicillin treatment
Source: BMC Microbiol. 2016 Sep 6;16(1):205. doi: 10.1186/s12866-016-0823-3 (PMC5012095; doi:10.1186/s12866-016-0823-3)
Supplement: Additional file 1: Figure S1. — Bacterial counts over time for 12 individual competing strains in a pig intestine, represented by different colours (top-left). Sum of susceptible (black) and resistant (red) counts (top right). Mean with 95 % simulation envelope from 100 model repeats (bottom left). Mean fraction of resistant counts over time (bottom right). Figure S2. Mean resistance fraction with 95 % simulation error bars from 100 model repeats at three different time points: day 0, day max and day 35. Day max represents the day with the maximum resistance. Different colours represent dosing frequencies and the columns represent treatment durations. Figure S3. Bacterial counts over time for 12 competing strains (different colours) with different growth characteristics without treatment (left), and with treatment (right). Vertical dotted lines indicate treatment duration. (PDF 466 kb) [file 12866_2016_823_MOESM1_ESM.pdf]

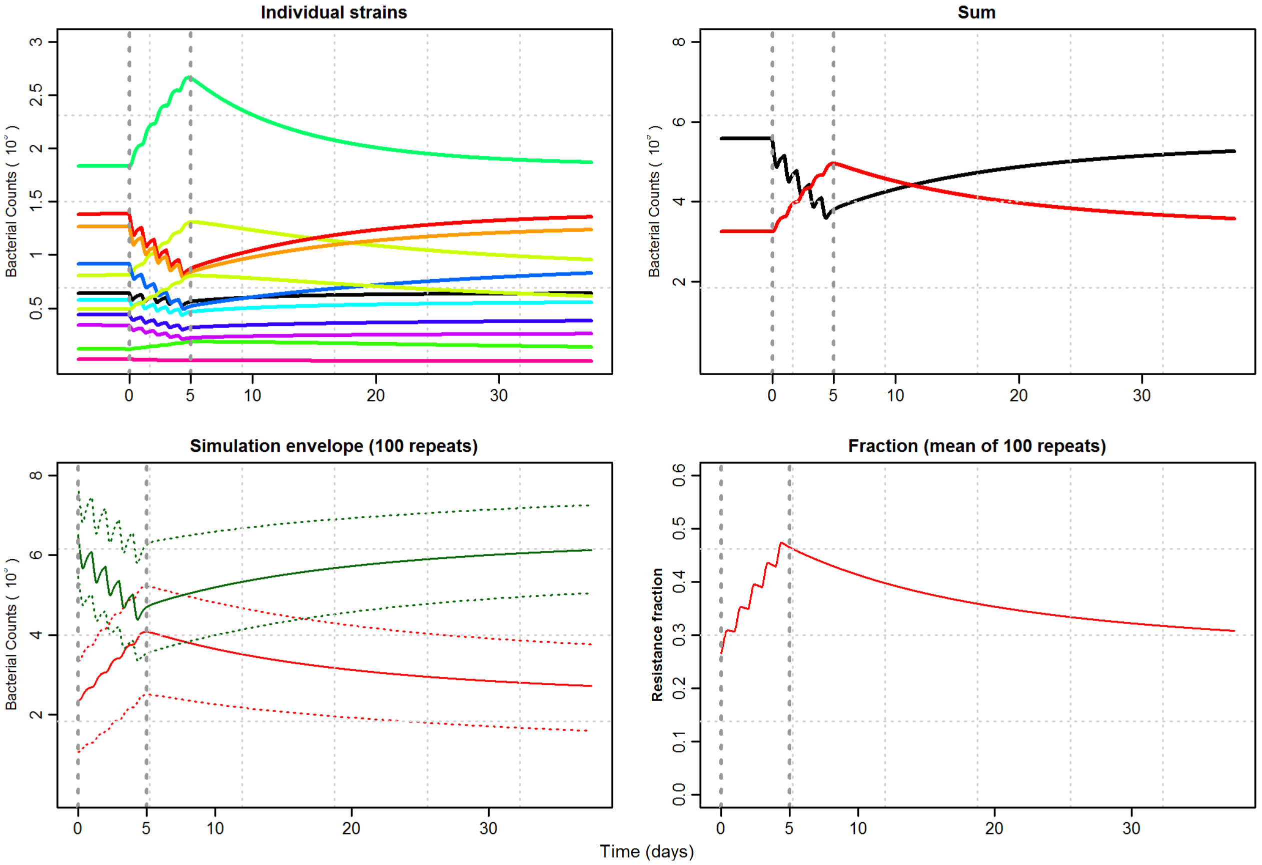

Supplementary figure S1: Bacterial counts over time for 12 individual competing strains in a pig intestine, represented by different colours (top-left). Sum of susceptible (black) and resistant (red) counts (top right). Mean with 95% simulation envelope from 100 model repeats (bottom left). Mean fraction of resistant counts over time (bottom right).

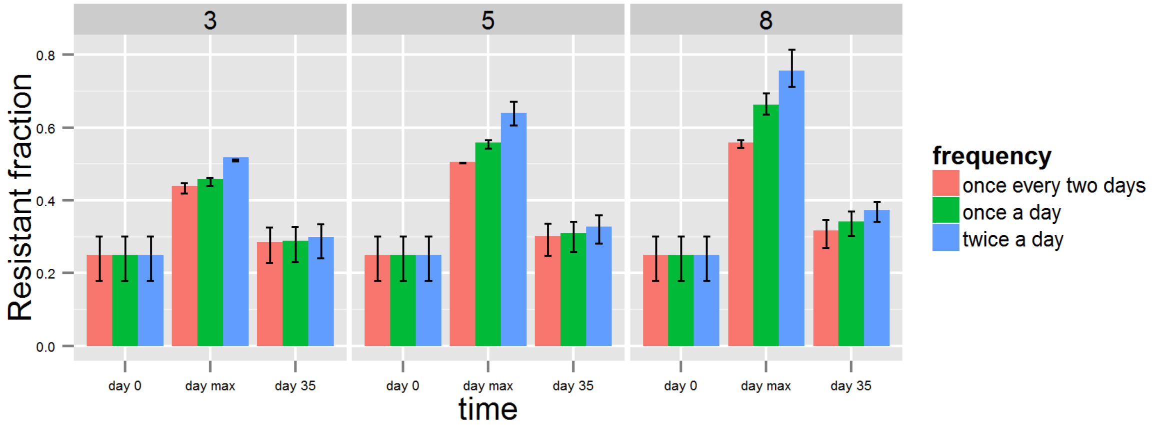

Supplementary figure S2: Mean resistance fraction with 95% simulation error bars from 100 model repeats at three different time points: day 0, day max and day 35. Day max represents the day with the maximum resistance. Different colours represent dosing frequencies and the columns represent treatment durations.

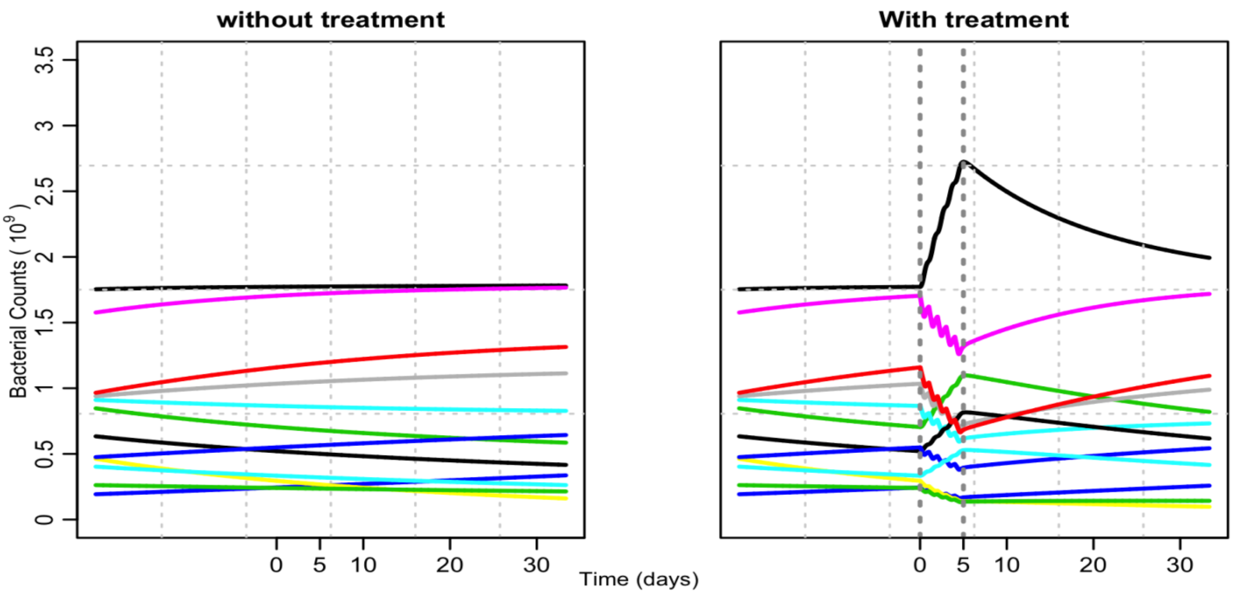

Supplementary figure S3: Bacterial counts over time for 12 competing strains (different colours) with different growth characteristics without treatment (left), and with treatment (right). Vertical dotted lines indicate treatment duration.
